# A Simply Equipped Fourier Ptychography Platform Based on an Industrial Camera and Telecentric Objective

**DOI:** 10.3390/s19224913

**Published:** 2019-11-11

**Authors:** Shaohui Zhang, Guocheng Zhou, Ying Wang, Yao Hu, Qun Hao

**Affiliations:** School of Optics and Photonics, Beijing Institute of Technology, Beijing 100081, China; guocheng_zhou@foxmail.com (G.Z.); wy365i@163.com (Y.W.); huy08@bit.edu.cn (Y.H.); qhao@bit.edu.cn (Q.H.)

**Keywords:** fourier ptychography, microscope platform, computational imaging, phase retrieval

## Abstract

Fourier ptychography microscopy (FPM) is a recently emerged computational imaging method, which combines the advantages of synthetic aperture and phase retrieval to achieve super-resolution microscopic imaging. FPM can bypass the diffraction limit of the numerical aperture (NA) system and achieve complex images with wide field of view and high resolution (HR) on the basis of the existing microscopic platform, which has low resolution and wide field of view. Conventional FPM platforms are constructed based on basic microscopic platform and a scientific complementary metal–oxide–semiconductor (sCMOS) camera, which has ultrahigh dynamic range. However, sCMOS, or even the microscopic platform, is too expensive to afford for some researchers. Furthermore, the fixed microscopic platform limits the space for function expansion and system modification. In this work, we present a simply equipped FPM platform based on an industrial camera and telecentric objective, which is much cheaper than sCMOS camera and microscopic platform and has accurate optical calibration. A corresponding algorithm was embedded into a conventional FP framework to overcome the low dynamic range of industrial cameras. Simulation and experimental results showed the feasibility and good performance of the designed FPM platform and algorithms.

## 1. Introduction

The field of view (FOV) and spatial imaging resolution of a microscopic platform contradict each other. Space–bandwidth product (SBP) [1], the product of FOV and spectral range of the signal entering the optical system, is an important parameter reflecting the throughput of a microscopic system. Larger SBP is highly desirable for the increasing requirements of biomedical applications. An effective conventional method to increase SBP is imaging the sample with a high numerical aperture (NA) objective and translating the sample laterally to increase the FOV. However, the mechanical method requires precise control over actuation, optical alignment, motion tracking, and feedback.

Fourier ptychography microscopy (FPM) [2,3,4,5,6,7] is a major branch of computational microscopy, which makes full use of optimization algorithms and inverse problem-solving algorithms to overcome the physical limitations of system hardware. In a typical FPM platform, a light-emitting diode (LED) array is utilized as the light source to provide angle-varied illuminations, and a scientific complementary metal–oxide–semiconductor (sCMOS), which has ultrahigh dynamic range and ultralow noise, is equipped to sequentially capture different frequency components of the sample. FPM shares its roots with optical synthetic aperture [8,9,10] and phase retrieval algorithms [11,12,13,14]. In the FPM imaging process, the sample is sequentially illuminated by each LED located on different positions of the LED array, and a series of corresponding low-resolution (LR) images are captured simultaneously. Then, the FPM algorithm iteratively stitches the low-resolution images together in Fourier domain to recover an accurate, complex high-resolution (HR) and high-SBP image of the sample.

Synthetic aperture requires accurate knowledge of the positions and shape of the corresponding spectrum of each low-resolution image. Therefore, the relative position and azimuth between the LED array, sample, objective, tube lens, and camera need precise calibration. Considerable work has been conducted on the calibration process and adaptive correction methods to recover the system key parameters [6,15,16,17]. Usually, the coaxiality of the objective and the tube lens is precalibrated by the manufacturer, and users lack the capability to adjust this degree of freedom. However, some low-end microscopes have poor coaxiality between the objective and the tube lens, which will not only have significant impact on conventional microscopic imaging but will also seriously decrease the quality of the reconstructed HR image for FPM systems. It is therefore difficult to implement a FPM platform based on a low-end microscope.

The amplitude difference between low- and high-frequency components is very large [18,19,20]. A sCMOS sensor is utilized to improve signal-to-noise ratio, expand the dynamic range of acquisition, and obtain higher spectrum information of the sample. Although sCMOS has excellent performance, it is also very expensive and unaffordable for some researchers.

In this work, we designed and implemented a simply equipped FPM platform using an industrial camera and telecentric objective lens. A corresponding FPM algorithm for this system was used to overcome the problem of low dynamic range and high noise of the industrial camera. The remainder of this paper is organized as follows. We introduce the conventional FPM setup and algorithm framework in Section 2. In Section 3, the scheme of the designed FPM platform is presented. In Section 4.1, we show a system alignment method based on image characteristics. In Section 4.2, we present some representative results obtained with the built FPM system and discuss and evaluate the performance of the system. Finally, the conclusions are summarized in Section 5.

## 2. Principle of Fourier Ptychography Microscopy

**Figure 1:**
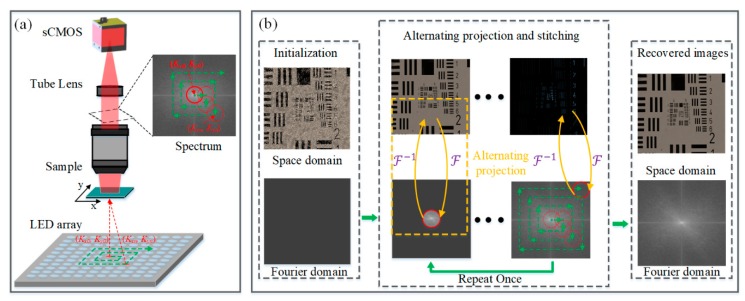
(**a**) Fourier ptychography microscopy (FPM) setup and (**b**) FPM algorithm framework.

The conventional FPM setup and algorithm framework are shown in Figure 1. The essential difference between a general optical microscope and a FPM platform is the construction method of the light source. Unlike usual microscopic light sources, FPM utilizes a LED array to illuminate the sample from multiple angles. The raw data collection procedure of FPM is straightforward. For each LED illumination, a LR image is recorded. The filtering function of the objective lens in Fourier domain is considered to be a circular pupil, whose radius is determined by its NA and wavelength of the illumination light. Thus, each LR image corresponds to a subspectral region, and the shift amount of each subspectral relative to the center of the whole spectrum depends on the incident angle of the inclined plane wave. The FPM algorithm integrates alternating projection phase retrieval and synthetic aperture imaging and stitches different subspectral regions together in Fourier space to generate a HR spectrum. Finally, the HR image can be directly processed by Fourier transform of the HR spectrum.

The core of the FPM algorithm is phase retrieval inverse problem, which refers to recovering the missing phase information according to acquired amplitude information and some prior constraints. The relationship of a complex signal and its Fourier transform can be expressed as follows:

(1) $$\left|F\left(u,v\right)\right|exp\left[j\psi \left(u,v\right)\right]=ℱ\left\{f\left(x,y\right)\right\}=\iint_{-\infty }^{\infty }f\left(x,y\right)exp\left[-j2\pi \left(x\cdot u+y\cdot v\right)\right]dxdy$$

where (x,y) and (u,v) are the coordinates in the space and frequency domain, respectively. Thus, the problem of phase retrieval can be mathematically stated as follows: Given the intensity measurement of a signal’s Fourier transform $I\left(u,v\right)={\left|F\left(u,v\right)\right|}^{2}$, which is equivalent to the Fourier transform of a signal’s autocorrelation, and some prior constraints about the signal f(x,y)=|f(x,y)|exp[jφ(x,y)], such as space size constraint, the missing phase part exp[jψ(u,v)] can be recovered.

The FPM algorithm shares its roots with conventional phase retrieval problem, but it can effectively alleviate its ambiguities, such as the twin-image problem. FPM algorithm is mainly divided into six steps [2]:

(1) Start with an initial guess at the HR spectrum $\Psi_{0}\left(k_{x},k_{y}\right)$, where $\left(k_{x},k_{y}\right)$ represents the coordinates in Fourier domain.

(2) Select a subregion of the initial spectrum and apply Fourier transformation to generate a LR target image. The subregion spectrum and corresponding LR image can be expressed as

(2) $$\psi_{0}\left(k_{m},k_{n}\right)=\Psi_{0}\left(k_{x},k_{y}\right)\cdot P\left(k_{x}-k_{m},k_{y}-k_{n}\right)$$

(3) $$\sqrt{I_{l\left(m,n\right)}}e^{\varphi l}=F^{-1}\left[\psi_{0}\left(k_{m},k_{n}\right)\right]$$

where the low-pass filter $P\left(k_{x}-k_{m},k_{y}-k_{n}\right)$ indicates the function of objective, and $\sqrt{I_{l\left(m,n\right)}}$ and $e^{\varphi l}$ are the amplitude and phase part of the LR image, respectively. The subscript *l* denotes low resolution, while *m* and *n* indicate the LED index along x and y axes, respectively.

(3) Replace the amplitude component of the LR target image $\left|\sqrt{I_{l\left(m,n\right)}}\right|$ with the square root of the obtained corresponding LR image $\sqrt{I_{m\left(m,n\right)}}$ and generate an updated LR image $\sqrt{I_{m\left(m,n\right)}}e^{\varphi l}$, where the subscript *m* indicates measurement.

(4) Then, apply Fourier transformation and circular low-pass filter to the updated LR image and obtain an undated subregion spectrum:

(4) $$\psi_{0}\left(k_{m},k_{n}\right)=F\left[\sqrt{I_{m\left(m,n\right)}}e^{\varphi l}\right]\cdot P\left(k_{x}-k_{m},k_{y}-k_{n}\right).$$

(5) Repeat steps 2–3 until all subregions in the spectrum have been updated. Then, a new HR spectrum $\Psi_{0}\left(k_{x},k_{y}\right)$ is obtained.

(6) Repeat steps 2–4 until a self-consistent solution is achieved. The final reconstructed HR image can be obtained by applying Fourier to the final HR spectrum.

## 3. System Design and Implementation

**Figure 2:**
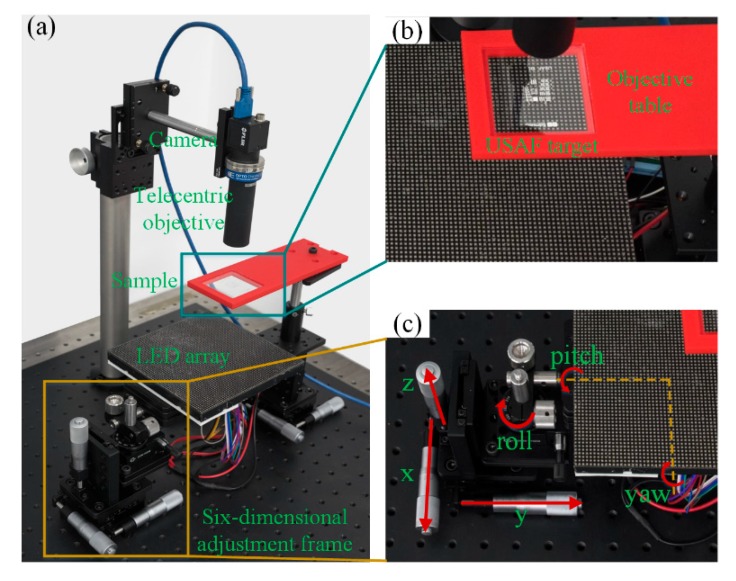
The implemented FPM platform. (**a**) The framework and basic components. (**b**,**c**) Enlargement details of the subregions in (**a**).

We designed and implemented a simply equipped FPM platform with an industrial camera and telecentric objective. The designed physical platform is illustrated in Figure 2a. In our system, a LED array (CMN, P 2.5, 31 × 31, emission wavelength λ = 633 nm) is mounted on a six-dimensional (6-D) adjustment frame, which is used to change the position and attitude angle of the LED array relative to the sample, telecentric objective, and industrial camera. The 6-D adjustment frame is composed of two one-dimensional linear displacement tables (DCH, GCM-TP13ML, adjustment range ±6.5 mm, fine-tuning resolution 10 μm), a lifting displacement table (DCH, GCM-V25M, adjustment range 25 mm, fine-tuning resolution 10 μm), a one-dimensional rotary table (DCH, GCM-1102M, adjustment range ±5°, fine-tuning resolution ±10′), and a two-dimensional angle adjustment frame (Thorlabs, KM200B/M, adjustment range ±3°, adjuster thread 1/4″−80). We used a data acquisition card (Advantech, USB-4751L) that has a series of output channels to drive the LED array with pulse width modulation (PWM) signal. The PWM drives each LED unit by cycling the current on and off rapidly at a fixed frequency with predesigned duty cycle. The luminous intensity of LED can be achieved by integrating the PWM signal. A 3-D printed objective table is fixed on a 3-D adjusting frame. The microscopic optics, consisting of objective and tube lens in conventional microscopic platform, is replaced by a telecentric objective (OPTO, TC23004, magnification 2x, NA ≈ 0.1, FOV 4.25 mm × 3.55 mm, working distance 56 mm). An industrial camera (Flir, BFS-U3-31S4C-C, sensor size 1/1.8", dynamic range 50 dB, pixel size 3.45 μm) is used to acquire and record sequential raw low-resolution images. The combination of industrial camera and telecentric objective can be adjusted in height by a 1-D guideway. The abovementioned FPM platform has sufficient adjustment freedom to ensure the relative position and attitude angle between LED array plane, camera plane, and optical axis of objective to meet the requirements of FPM algorithm on system parameters. Figure 2b presents an enlargement of the objective table and the USAF target in Figure 2a. The distance between the objective and sample is about 56 mm, which is much larger than conventional microscopic objectives. Thus, there is enough room to add necessary devices to achieve functional expansion. Figure 2c shows an enlargement of the 6-D adjustment frame in Figure 2a. We integrated one 3-D translation platform, one 1-D rotary table, and one 2-D angle adjustment frame to form a 6-D adjustment frame.

Using an industrial camera instead of sCMOS camera can greatly reduce the cost of constructing a FPM platform. However, compared to an industrial camera, sCMOS has excellent dynamic range (almost 90 dB), quantum efficiency (≥80%), and readout noise (<2.1e^_^), which is more powerful for obtaining high-frequency components with small amplitude. In our proposed framework, to cover the range of low- and high-frequency components of the sample, we expanded the dynamic range of the collected signal by adjusting the luminous intensity of LED and exposure time of the industrial camera simultaneously. For LED units in the middle, corresponding to low-frequency components of the sample, we used a PWM signal with low duty cycle as the driving signal. Meanwhile, the industrial camera was set to a relatively lower exposure time. For LED units located outside, high duty cycle and relatively higher exposure time were used to increase the signal-to-noise ratio of the captured images.

FPM combines the concept of synthetic aperture, so the position and shape information of each subaperture needs to be known very accurately. Therefore, alignment is a critical process in the construction of the FPM platform. As mentioned above, the optical axis of the telecentric objective was normal to the CMOS plane of the industrial camera; this was guaranteed by the mechanical chuck. Therefore, the alignment process of the proposed platform could be realized by only adjusting the six degrees of freedom of the LED array. We utilized the image characteristics of the aperture diaphragm as feedback and criterion for precision mechanical adjustment of the 6-D adjustment frame, as shown in Figure 2c.

The low-resolution image acquisition process of our designed system is the same as conventional FPM platform. The LED unit in the array is sequentially turned on in a predesigned order and acquires corresponding low-resolution images simultaneously. In conventional FPM platform and algorithms, the illumination intensity onto the sample is considered to be consistent during the process of turning on different LED units sequentially. However, both the relative distance and angle between each LED unit and the sample varies, leading to different illumination intensities onto the sample, even when the luminous intensity of each LED is the same. Moreover, the luminous intensity and model of each LED will inevitably have some difference in the manufacturing process. Besides the nonideal LED illumination characteristics, the response characteristics of the image sensors are not strictly linear either. Thus, both the inconsistent illumination of a plane LED array and nonlinear response of the image sensor will cause reconstruction error during the phase retrieval process in FP algorithm. In order to solve the problem, we need to know the accurate luminous model and intensity characteristics of the LED and response characteristics of the camera, which is difficult and time-consuming. In our proposed FP framework, we utilized an adaptive FPM algorithm to acquire the real illumination information and improve the reconstruction quality.

## 4. Experiments

### 4.1. System Alignment

**Figure 3:**
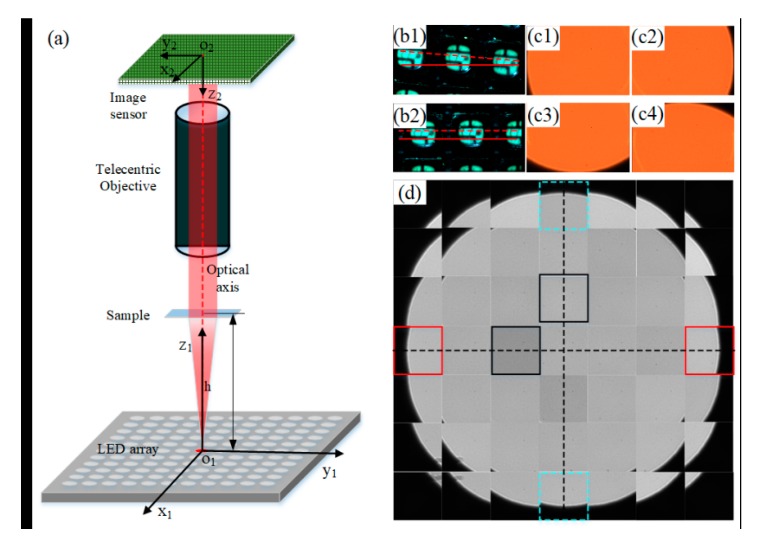
Alignment of proposed FPM platform. (**a**) The coordinate systems based on complementary metal–oxide–semiconductor (CMOS) plane and light-emitting diode (LED) array plane. (**b1,b2**) The criterion for roll angle adjustment of the LED array. (**c1**–**c4**) The image criterion for adjustment of central LED position. (**d**) A mosaic image combining raw images of aperture diaphragm containing bright-field components.

Similar to conventional ptychography [21,22,23], which has strict alignment requirements, position and angle errors are of great importance in the FPM framework. This is because the wave vector of each incident plane wave is determined by the relative position of each LED to the sample. Two orthogonal directions of LED arrangement on the array should be parallel to those of pixel arrangement on the image sensor plane. Moreover, the optical axis of the objective should pass through the center of the image sensor plane and the LED array plane and perpendicular to the two planes. In our implemented FPM platform, the geometric relationship between the optical axis of telecentric objective and camera sensor plane is guaranteed by mechanical chuck. Therefore, alignment could be achieved by only adjusting the six degrees of freedom of the LED array, including three degrees of freedom in axis (x, y, z) and three degrees of freedom in angle (yaw, pitch, roll). As shown in Figure 3a, the coordinate axes depending on the image sensor plane and the corresponding ones depending on the LED array plane should be parallel to each other. Both coordinate origins O1 and O2 should be on the optical axis of the telecentric objective.

As there are multiple variables involved in the alignment process, we took a divide-and-conquer approach that adjusted each degree of freedom sequentially. The order of alignment was roll angle adjustment, LED central position adjustment, and pitch and yaw angle adjustment. In the whole system alignment process, no samples were needed.

First, we adjusted the height of the LED array to get a clear image of its segment, which contained several LED units. As shown in Figure 3b, we took the parallelism between the direction of the pixel array and the LED image as the criterion for adjusting roll angle. Figure 3b1,b2 shows two cases in the process of roll angle adjustment, in which the solid and dotted lines represent O2X2 and O1X1 in Figure 3a, respectively.

Second, the LED array was adjusted away from the image sensor to its final position, and the central LED was adjusted on the optical axis of the telecentric objective with the help of the edge of the aperture diaphragm on the image as the criterion. Figure 3c1–c4 presents four critical positions during the central LED position alignment process by adjusting the 2-D translation stage [6]. Figure 3c1,c2, in which the image of the aperture diaphragm is tangent to the left and right edge of the image sensor, respectively, corresponds to two critical stage positions x1 and x2 in the x direction. Similarly, Figure 3c3,c4 corresponds to two critical stage positions y1 and y2 in the y direction. Finally, we adjusted the stage to the coordinate of ((x1+x2)/2, (y1+y2)/2) to complete the adjustment of the central position of the LED array.

Lastly, by utilizing the symmetrical characteristic of the images as criterion, we could achieve the relative yaw and pitch alignment of the LED array. A series of LED were turned on sequentially, and the corresponding aperture diaphragm images were recorded. Figure 3d shows the mosaic of multiple images corresponding to different LEDs. If the LED array is not parallel to the image sensor plane, the subimages at symmetrical positions are asymmetrical to each other. The image pairs inside the red solid boxes and dotted green boxes in Figure 3d indicate that the yaw and pitch angles of the LED array had been adjusted to the right position. In Figure 3d, besides the symmetry information between the subimages, one can also find the brightness difference among subimages (surrounded by black frames), which refers to inconsistent illumination or image brightness response corresponding to each low-resolution image.

### 4.2. Experimental Verification

**Figure 4:**
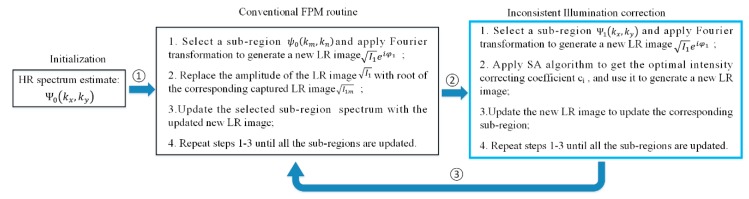
A flow chart of the simulated annealing (SA)-embedded FPM algorithm.

For experimental demonstration, on the basis of system alignment and cooperative control software for LED array and industrial camera, we characterized the resolution performance of the implemented FPM system by imaging USAF target and biological samples. The distance between the sample and LED array was adjusted to 93 mm to ensure the overlap ratio between adjacent subspectral regions met the phase retrieval criterion [19]. We illuminated the USAF target with 31 × 31 LEDs (spacing 2.5 mm) sequentially and captured 961 corresponding images with different exposure times. The illumination NA was about 0.37. The camera exposure time corresponding to central 7 × 7 LED array was set at 100 ms, while it was set at 150 ms for the five-ring LEDs around the central 7 × 7 array, 450 ms for six-ring LEDs around the central 19 × 19 array, and 700 ms for all the other ones. Then, the captured LR images were stitched together in Fourier domain with phase retrieval algorithms. The main algorithm framework was constructed according to the literature [2,3]. As shown in Figure 4, in our HR image reconstruction process, simulated annealing algorithm was incorporated into conventional FP algorithm framework to correct the inconsistent illumination and light intensity response.

**Figure 5:**
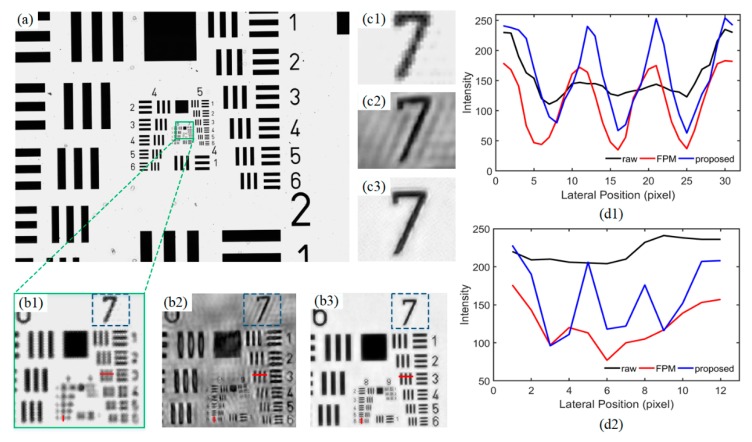
Resolution characterization of the implemented FPM platform. (**a**) The full field of view (FOV) of the USAF target recorded with the implemented platform. (**b1**–**b3**) An enlargement of the subregion in Figure (**a**), the reconstructed high-resolution (HR) intensity image with conventional FP algorithm, and the HR image recovered with SA-embedded FP algorithm, respectively. (**c1**–**c3**) Enlargements of the content inside the blue boxes in Figure (**b1**–**b3**). (**d1**–**d2**) Intensity profiles of the red lines (element 3, group 7 and element 5, group 8) shown in (**b1–b3**).

The LR bright-field image with full field of view captured with the implemented FPM platform is shown in Figure 5a. The spatial resolution was limited by the NA of the telecentric objective, which was about 0.1. Figure 5b1 shows an enlargement of the subregion inside the red square in Figure 5a. It can be seen that features smaller than group 7, element 3 could not be resolved in this subimage. The reconstructed HR image with conventional FP algorithm is shown in Figure 5b2, in which the resolution characteristic was obviously improved compared to that in the original LR image. Nevertheless, the background of the HR image with conventional FP algorithm was inhomogeneous, and obvious stripes were clearly observable in some subregions. As mentioned earlier, both the illumination and image acquisition process will introduce errors. The nonideal reconstruction result in Figure 5b2 might have been due to the inconsistent illumination and exposure time during the capture of each raw LR image. To correct the errors in reconstruction and improve the HR quality, we incorporated stimulated annealing (SA) algorithm into conventional FP algorithm to adaptively correct the brightness of each LR image. The reconstructed HR image with SA-embedded FP algorithm is shown in Figure 5b3, in which the features in group 9, element 3 could be clearly resolved. In addition, the clarity of the image background was also improved. Figure 5c1–c3 presents the contrast results of the reconstructed image background corresponding to raw image (shown in Figure 5b1), recovered image with conventional FPM (shown in Figure 5b2), and the image with the proposed algorithm (shown in Figure 5b3), respectively. It can be seen that, for raw data sets with different exposure times, the conventional FPM could actually increase the resolution. However, there were ripples in the blank background region. The ripples in the HR image could be removed with our improved FPM algorithm. Intensity profiles of the lines shown in red (element 3, group 7 and element 5, group 8) in Figure 5b1–b3 are displayed in Figure 5d1,d2. It can be noted that the intensity profiles of the USAF chart in the raw image was relatively flat and irregular, while the profiles reconstructed with conventional FPM and the proposed algorithm exhibited obvious peaks and valleys that were much closer to the true value. A comparison of the results indicated that the designed and implemented FPM platform could reduce the noise in the background and achieve a lateral resolution beyond 2.19 μm (the size of element 5, group 8 was 465 pairs/mm).

**Figure 6:**
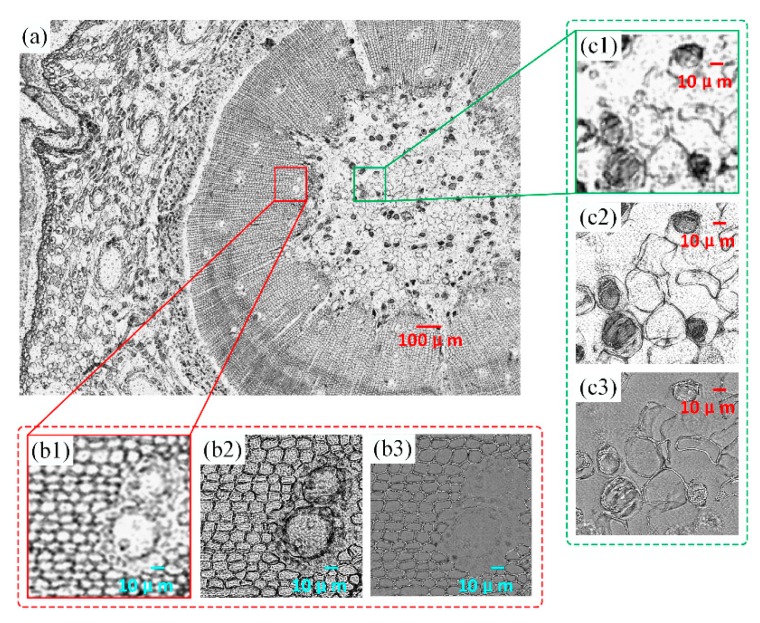
Experiment results of a pine stem transection specimen. (**a**) The full FOV of the pathology sample recorded with the implemented platform. (**b1**–**b3**) Enlargement, reconstructed HR intensity image, and reconstructed phase image, respectively, of a small segment located inside the red box in the original captured image. (**c1**–**c3**) Enlargement, reconstructed HR intensity image, and reconstructed phase image, respectively, of another small segment located inside the green box in the original captured image.

We also verified our platform and algorithm with a pine stem transection specimen (SAGA). The full field of view is shown in Figure 6a. Image comparison results of two subregions in the slide are presented in Figure 6b,c. Figure 6b1,c1 shows the enlargement of the original raw LR image, while Figure 6b2,b3,c2,c3 show the corresponding reconstructed HR intensity and phase parts. The resolution of the chosen region was obviously improved with the implemented platform and algorithm. The cell wall and internal structure of the pine stem could be clearly resolved in the HR images. Meanwhile, the phase distribution information was also recovered simultaneously. In most cases, biological samples are transparent and therefore cannot be seen directly with conventional microscopy. The results shown in Figure 6b3,c3 indicates that this custom FPM platform can achieve qualitative phase measurement.

## 5. Conclusions

We designed and implemented a simply equipped FPM platform with an industrial camera and telecentric objective. The telecentric objective, corresponding to microscopic objective and tube lens in conventional microscopic platform, has a concise and simple structure and much longer working distance. Because of its simple and flexible structure, the platform can be easily extended and modified. The alignment method of the proposed system was studied and found to be effective. We utilized the image characteristics of the LED array and systematic aperture diaphragm as criteria to achieve mechanical alignment of the designed FPM platform. In order to compensate the small dynamic range and low signal-to-noise ratio of the industrial camera, we changed the illumination intensity of the LED and exposure time of the camera simultaneously for LED located at different positions. In the reconstruction process, simulated annealing algorithm was incorporated into conventional FP algorithm to correct the illumination model error and inconsistency of the luminous intensity of each LED.

To evaluate the FPM platform we have built, USAF resolution chart and biological samples were used. We compared the original LR image, the reconstructed HR intensity distribution of the sample with conventional FPM algorithm, and HR image recovered with SA-embedded FP algorithm. The performance of the designed and implemented FP platform was comparable to that based on conventional microscopic platform and sCMOS. Meanwhile, the total price of constructing this proposed FPM platform is less than US$1500, which is much cheaper than an ordinary microscope (Olympus BX43, US$7000) and sCMOS camera (pco.edge 4.2, US$19,000). Furthermore, the proposed custom-built setup is expandable to add more functionality (polarized-light microscopy, light-field microscopy, etc.).
